# TBK1 Protects Vacuolar Integrity during Intracellular Bacterial Infection

**DOI:** 10.1371/journal.ppat.0030029

**Published:** 2007-03-02

**Authors:** Andrea L Radtke, Laura M Delbridge, Siddharth Balachandran, Glen N Barber, Mary X. D O'Riordan

**Affiliations:** 1 Department of Microbiology and Immunology, University of Michigan Medical School, Ann Arbor, Michigan, United States of America; 2 Department of Microbiology and Immunology, University of Miami School of Medicine and Sylvester Comprehensive Cancer Center, Miami, Florida, United States of America; Yale University, United States of America

## Abstract

TANK-binding kinase-1 (TBK1) is an integral component of Type I interferon induction by microbial infection. The importance of TBK1 and Type I interferon in antiviral immunity is well established, but the function of TBK1 in bacterial infection is unclear. Upon infection of murine embryonic fibroblasts with *Salmonella enterica* serovar Typhimurium *(Salmonella),* more extensive bacterial proliferation was observed in *tbk1^−/−^* than *tbk1^+/+^* cells*.* TBK1 kinase activity was required for restriction of bacterial infection, but interferon regulatory factor-3 or Type I interferon did not contribute to this TBK1-dependent function. In *tbk1^−/−^*cells, *Salmonella,* enteropathogenic *Escherichia coli,* and Streptococcus pyogenes escaped from vacuoles into the cytosol where increased replication occurred, which suggests that TBK1 regulates the integrity of pathogen-containing vacuoles. Knockdown of *tbk1* in macrophages and epithelial cells also resulted in increased bacterial localization in the cytosol, indicating that the role of TBK1 in maintaining vacuolar integrity is relevant in different cell types. Taken together, these data demonstrate a requirement for TBK1 in control of bacterial infection distinct from its established role in antiviral immunity.

## Author Summary

Early control of invading microbial pathogens is an essential function of the host response to infection. Previous studies have shown that upon viral infection, a protein called TANK-binding kinase-1(TBK1) signals the induction of a program of protection that results in inhibition of viral replication. During infection of mammalian cells by bacteria, a different type of microbe than a virus, TBK1 also sends signals, but the functional contribution of TBK1 to controlling bacterial infection was unknown. Here, we show that TBK1 does protect host cells from bacterial infection; however, the TBK1-dependent mechanisms that inhibit viral infection were not effective against bacterial growth. Instead, TBK1 maintained the integrity of the vacuolar compartment, consisting of small membrane-bound vesicles, where the invading bacteria were trapped. In the absence of TBK1, pathogens such as *Salmonella,* enteropathogenic *Escherichia coli,* and Group A *Streptococcus* were able to escape from the confining host vacuoles and grow to high levels within the host cytosol. Thus, TBK1 plays an important role in the cellular response to bacterial infection, distinct from its function in antiviral immunity.

## Introduction

Host organisms employ a multitude of innate defense mechanisms against invading microbial pathogens. Functions of the innate immune system include control and destruction of pathogens and instruction of the developing adaptive immune response through expression of cytokines, chemokines, and other proinflammatory molecules [1,2]. Innate recognition of invading pathogens can occur through pattern recognition receptors, such as Toll-like receptors (TLRs), which bind to molecules characteristic of microbial organisms like lipopolysaccharide (LPS) [3]. Two well-characterized signaling pathways associated with TLR stimulation are the MyD88-dependent pathway, which primarily results in NFκB activation, and the TANK-binding-kinase-1 (TBK1)-dependent pathway that induces transcription of Type I interferon genes [4]. The importance of the MyD88-dependent pathway in antibacterial immunity is well established, but the functional contribution of TBK1-dependent signaling in protecting against bacterial infection is unknown [3].

TBK1, also termed NFκB-activating kinase or TRAF2-associated kinase, is a ubiquitous member of the IκB kinase (IKK) family that is required for embryonic development [5–7]. Upon microbial infection or LPS treatment, TBK1 phosphorylates the transcription factor interferon regulatory factor-3 (IRF3), resulting in IRF3 translocation into the nucleus and transcription of target genes, such as *ip10* (IFN-gamma inducible protein 10), *ifnb* (interferon beta; a Type I interferon), and subsequent interferon response genes such as *mx1* [8–10]*.* TBK1 is required for optimal induction of *ifnb* and Type I interferon-dependent antimicrobial effector mechanisms during viral infection [11]. Induction of TBK1-dependent signaling by viruses has been extensively studied and occurs upon viral recognition by TLRs or upon cytosolic binding of viral double-stranded RNA by the DEAD-box helicases, RIG-I and MDA5 [12–16]. Gram-negative bacterial infections can also trigger TBK1-dependent interferon induction by association of LPS with the TLR4 signaling complex [10,17]. In contrast, extracellular Gram-positive bacteria do not activate expression of interferon genes, although cytosolic Gram-positive bacteria can upregulate *ifnb* transcription in an IRF3-dependent manner [18,19].

To investigate the requirement for TBK1 in response to bacterial infection, we used Salmonella enterica serovar Typhimurium as a model Gram-negative bacterium. *Salmonella* is a facultative intracellular pathogen that replicates within macrophages and non-phagocytic cells [20,21]. *Salmonella* invades non-phagocytic cells by secreting effector proteins through a syringe-like Type III secretion system (T3SS) encoded on *Salmonella* pathogenicity island-1 (SPI-1) to induce membrane ruffling and bacterial uptake [22]. After entry into the host cell, *Salmonella* resides in a membrane-bound compartment termed the *Salmonella-*containing vacuole (SCV). Subsequent to invasion, the SCV progresses through early stages of endocytic maturation and acquires the late endosomal marker, lysosomal-associated membrane protein 1 (LAMP-1) [23]. At this stage, the SCV can diverge from the endocytic pathway by avoiding lysosome fusion and can localize to the perinuclear region [24]. Many *Salmonella* remain in the SCV and replicate; however, a small percentage of bacteria escape into the host cytosol where they acquire host-derived ubiquitin [25].

Like viruses, intracellular bacterial pathogens exploit the host cell as a replicative niche. By analogy to known antiviral immune mechanisms, we hypothesized that TBK1 would also protect host cells from infection by intracellular bacterial pathogens. However, specific effector mechanisms of antiviral innate immunity, such as Type I interferon-dependent induction of 2–5′ oligoadenylate synthase, which facilitates viral RNA degradation, would not necessarily be effective against intracellular bacteria [26]. Since loss of TBK1 results in embryonic lethality, we used *tbk1^+/+^* and *tbk1^−/−^* mouse embryonic fibroblasts (MEFs) or RNAi to test the requirement for TBK1 during bacterial infection. Here, we show that TBK1 mediates an early cellular response to infection by *Salmonella* and other bacteria by maintaining these pathogens in a restrictive vacuolar compartment.

## Methods

### Bacterial Strains and Cell Culture

Strains used in this study are described in Table S1. *Salmonella* strains expressing green fluorescent protein (GFP) were constructed by electroporating the bacteria with pFPV25.1 obtained from C. Detweiler [27]. The *Salmonella* strain expressing red fluorescent protein (RFP) was constructed by amplifying the RFP gene from pGEM:RFP (J. A. Bauer, University of Michigan Medical School), ligating the sequence into PCR-II-Topo (Invitrogen, http://www.invitrogen.com), and then electroporation into *Salmonella.* MEFs were obtained from W. C. Yeh (*tbk1* ^+/+^ and *tbk1* ^−/−^) and K. Mossman and B. Williams (*irf3* ^+/+^ and *irf3* ^−/−^). MEFs and RAW264.7 cells (American Type Culture Collection) were grown in RPMI medium supplemented with 10% fetal bovine serum and 1% L-glutamine at 37 °C in 5% CO_2_. Experiments in *tbk1* ^+/+^ and *tbk1* ^−/−^ MEFs were reproduced both with primary MEFs in early passage and with MEFs that were immortalized by continuous culture and clonally derived. HeLa cells (ATCC) were grown in MEMα medium supplemented with 10% fetal bovine serum, 1% L-glutamine, 1% nonessential amino acids, and 1% sodium pyruvate at 37 °C in 5% CO_2_.

### Infections

All *Salmonella* strains were grown overnight in LB medium at 37 °C, shaking and back-diluted 1:100. When the bacteria reached exponential phase, they were washed twice with Dulbecco's PBS (D-PBS) and used to infect MEFs at a multiplicity of infection (m.o.i.) of 10 for 1 h, whereupon infected cells were washed three times with D-PBS and incubated in medium containing 100 μg/ml gentamicin for 2 h. The cells were then washed three times with D-PBS and fresh medium containing 5 μg/ml gentamicin was added. For intracellular growth curves, at indicated times post-infection (p.i.), three cover slips containing infected MEFs were removed and individually lysed in 5 ml sterile water; a fraction of the lysate was plated on LB agar to enumerate colony-forming units. *St* SPI-1^−^ required bystander infection with wild-type *Salmonella;* coinfections were performed with *Salmonella* grown and infected under the same conditions as stated for monotypic *Salmonella* infection in MEFs, except that the *St* SPI-1^−^ mutant was added at an m.o.i. of 100. Enteropathogenic Escherichia coli (EPEC) was grown statically overnight in LB at 25 °C, then back-diluted 1:100, and incubated shaking at 37 °C in serum-free DMEM for 1 h. EPEC were then used to infect MEF cells at an m.o.i. of 25 for 1 h; washing and gentamicin treatment were carried out as described for *Salmonella. Streptococcus pyogenes* were statically grown overnight at room temperature in BHI. The stationary phase bacteria were washed two times with PBS, vigorously vortexed, and used to infect MEFs at an m.o.i. of 25. The remainder of the infection was done under the same conditions as described for *Salmonella* infection in MEFs. Bacterial infections in transfected HeLa cells were performed as described for MEFs, except that *Salmonella* and S. pyogenes were incubated with the host cells for 30 min at an m.o.i. of 100 and EPEC at an m.o.i. of 50 for 1 h; the HeLa cells were washed three times with D-PBS and incubated with fresh medium for 30 min before gentamicin was added. Infections of RAW264.7 macrophages were performed with *Salmonella* at an m.o.i. of 10 for 30 min, EPEC at an m.o.i. of 50 for 45 min, and S. pyogenes at an m.o.i. of 25 for 30 min. The macrophages were washed three times with D-PBS, and medium with gentamicin added as described for MEFs. 2-μm beads were incubated with anti-streptavidin antibody in D-PBS + 1% BSA for 30 min, then washed three times in D-PBS + 1% BSA, added to the macrophage culture, and spun down onto the macrophage monolayer. Beads were incubated with the macrophages for 30 min and then the cells were washed three times with D-PBS before adding fresh medium.

### Transfections

HeLa cells or MEFs were re-suspended in the appropriate growth medium as described above and plated in a 6-well plate at a concentration of 5 × 10^5^ cells/plate. Cells were transfected for 24 h with either 4.2 μg of plasmid DNA or 40 μM of the indicated short interfering RNA (siRNA) (Ambion, http://www.ambion.com) complexed with Lipofectamine 2000 (Invitrogen, http://www.invitrogen.com). For siRNA experiments, transfected cells were trypsinized, split 1:2, and subjected to a second round of siRNA transfection as above, until time of harvest or infection with *Salmonella.* For transfection of RAW264.7 macrophages, cells were re-suspended in growth medium as described above and plated in a 6-well plate at a concentration of 1 × 10^6^cells/plate. Macrophages were transfected as described for HeLa and MEF cells.

### Microscopy

Cells were grown in appropriate medium in a 6-well plate at a concentration of 7 × 10^5^ cells/plate and infected as described above. At indicated times p.i., cover slips were removed and fixed in 3.7% paraformaldehyde in D-PBS. Cells were then washed three times with 0.1% Triton X-100 in PBS and blocked for 10 min with TBS-TX (25 mM Tris-HCl [pH 8.0], 150 mM NaCl, 0.1% Triton X100, 1% BSA). TBS-TX with primary antibody was added to the cells for 1 h. Cells were then rinsed three times with TBS-TX and incubated for 30 min with secondary antibody. Cover slips were rinsed with TBS-TX and mounted with Pro-Long Gold Antifade (Invitrogen). Samples were analyzed with the Olympus Fluoview FV-500 (http://www.olympus.com) confocal microscope using a 100× objective, unless otherwise stated, and Fluoview software. Quantitation of bacteria colocalized with LAMP-1 or ubiquitin was performed by scoring 150 randomly chosen bacteria per experiment for the presence of LAMP-1 or ubiquitin; only bacteria completely surrounded by LAMP-1 or ubiquitin as indicated by antibody staining were scored as colocalized. Transmission electron microscopy was performed on a Philips CM-100 transmission electron microscope equipped with automated compustage and Kodak 1.6 Megaplus (http://www.kodak.com) high-resolution digital camera. Samples were prepared as previously described [28].

### Antibodies and Reagents

The LAMP-1 (1D4B) rat monoclonal antibody was obtained from Santa Cruz Biotechnology (http://www.scbt.com), the anti-ubiquitin monoclonal antibody (FK2) from BIOMOL International (http://www.biomol.com), the anti-TBK1 monoclonal antibody from Imgenex (http://www.imgenex.com), and anti-streptavidin antibody from Molecular Probes (http://probes.invitrogen.com). TRITC-phalloidin was purchased from Invitrogen and 4′,6-Diamidino-2 phenylindole dihydrochloride (DAPI) from BioChemika (http://www.sigmaaldrich.com). Recombinant mouse interferon-β (PBL Biomedical Laboratories, http://www.pblbio.com) was used at 100 U/ml to treat cells overnight and throughout the course of infection. Lysotracker Red DND-99 (Invitrogen) was a gift from K. Collins (University of Michigan Medical School). Alpha-amanitin (BioChemika) was used at 50 μg/ml to pre-treat cells for 1 h prior to and during the course of infection. All siRNA reagents were obtained from Ambion. The EGFP-LC3 was obtained from Addgene, Incorporated (plasmid 11546) (http://www.addgene.com) and constructed in the laboratory of K. Kirkegaard. Beads and anti-streptavidin antibody were a gift from J. Swanson (2.01-μm streptavidin silicon oxide microspheres; Corpuscular, Cold Spring, New York, United States).

### Construction of TBK1-KD

The *tbk1* cDNA amplified from *tbk1^+/+^* MEF RNA was cloned into the expression vector pcDNA3 (Invitrogen). After sequence validation, QuikChange mutagenesis [29] was performed to replace lysine-38 with an alanine, which was validated by sequencing. TBK1:pcDNA3 and TBK1-KD:pcDNA3 both encoded a full-length TBK1 protein as determined by TNT Quick Coupled Transcription/Translation Systems (Promega, http://www.promega.com) and Western blot probed with anti-TBK1 antibody.

### Real Time Quantitative RT-PCR and Sequence Information

Total RNA was isolated from cells using the RNAeasy kit (Qiagen, http://www1.qiagen.com) and cDNA synthesis carried out using 2.5 μg of total RNA (M-MLV Reverse Transcriptase; Invitrogen). Real time RT-PCR analysis was performed with the MX3000p (Stratagene, http://www.stratagene.com) and Brilliant SYBR Green MasterMix (Stratagene). Relative amounts of cDNA were normalized to actin cDNA levels in each sample. The following primers were used for amplification: *ip10* (F- 5′ATGAGGGCCATAGG GAAGCTTGAA; R- 5′ACCAAGGGCAATTAGGACTAGCCA), *mx1* (F-5′ TTGTCTA CTGCCAGGACCAGGTTT; R-5′ TTTCAGGTGCTGGGTCATCTCAGT), *actin* (F-5′AGGTGTGATGGTGGGAATGG; R-5′GCCTCGTCACCCACATAGGA).

## Results

### TBK1 Restricts Intracellular Infection by *Salmonella*

**Figure 1:**
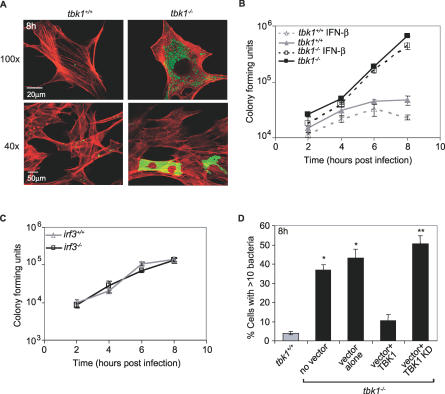
TBK1 Kinase Activity Suppresses *Salmonella* Intracellular Growth (A) MEFs were infected with *Salmonella*-GFP (green), fixed at 8 h p.i., counterstained with TRITC-phalloidin to visualize actin (red), and then analyzed by confocal immunofluorescence microscopy. Images were acquired using either a 100× or 40× objective. (B) *Salmonella* intracellular growth was measured over time in *tbk1^+/+^* (gray triangles) and *tbk1^−/−^* (black squares) MEFs in the absence (solid lines) or presence (dashed lines) of Type I interferon. Where indicated, MEFs were treated with 100 U/ml rIFN-β prior to and during infection. The data are representative of five independent experiments. (C) *Salmonella* intracellular growth in *irf3^+/+^* (gray triangles) and *irf3^−/−^* (black squares) MEFs was measured over time. The data are representative of three independent experiments. (D) MEFs were transfected with vector alone (pcDNA3), pcDNA3:TBK1, or pcDNA3:TBK1-KD where indicated in combination with pcDNA3:GFP as a marker for transfection. Percent infected cells that contained >10 *Salmonella,* out of 150 infected cells counted per experiment at 8 h p.i., was determined by fluorescence microscopy using DAPI to identify bacteria (*n* ≥ 3). Quantitation was limited to GFP-positive (transfected) MEFs. * and ** denote statistical significance of *p* ≤ 0.05 and 0.001, respectively, according to a one-tailed Student *t*-test comparing each *tbk1^−/−^* sample to the *tbk1^+/+^* control.

To investigate the role of TBK1 in the cellular response to bacterial infection, we infected *tbk1^+/+^* and *tbk1^−/−^* MEFs with *Salmonella* expressing GFP. *Salmonella* invaded the wild-type and mutant MEFs similarly; however, at 8 h p.i., the monolayer of *tbk1^−/−^* MEFs contained approximately 10-fold more bacteria than the wild-type MEFs (Figure 1A and 1B). Robust bacterial proliferation was observed in 35%–40% of infected *tbk1^−/−^* MEFs, which was consistent with immunofluorescence analysis of individual cells showing a more pronounced phenotype than that observed by measuring net increase in bacterial numbers. By immunofluorescence analysis, the remainder of the *tbk1^−/−^* infected MEFs appeared similar to *tbk1^+/+^* infected MEFs (Figure 1A; 40× magnification). Infected *tbk1^−/−^* MEFs that did not exhibit greater bacterial proliferation might have undergone an unproductive infection, since at 1 h p.i., 20%–30% of the bacteria in either wild-type or mutant MEFs were found in autophagosomes, and an additional 25%–30% in lysosomes, which are likely nonreplicative compartments (Figure S1A and S1B). The increased bacterial growth observed in *tbk1^−/−^* MEFs was not suppressed by addition of exogenous Type 1 interferon, nor was a similar phenotype observed when *Salmonella* infection was compared in *irf3^+/+^* and *irf3^−/−^* MEFs (Figures 1B, 1C, and S2A). Inhibition of de novo transcription and translation also had no effect on the phenotype (Figure S2B and S2C; unpublished data). However, the robust bacterial replication in *tbk1^−/−^* MEFs was substantially decreased by transient transfection with a plasmid expressing wild-type TBK1, but not a kinase dead mutant protein (TBK1 KD) [6] (Figure 1D). Together, these data indicate that TBK1 kinase activity limits intracellular infection of *Salmonella* independently of the IRF3-Type I interferon axis.

### *Salmonella* Escapes from the Endocytic Pathway in the Absence of TBK1

**Figure 2:**
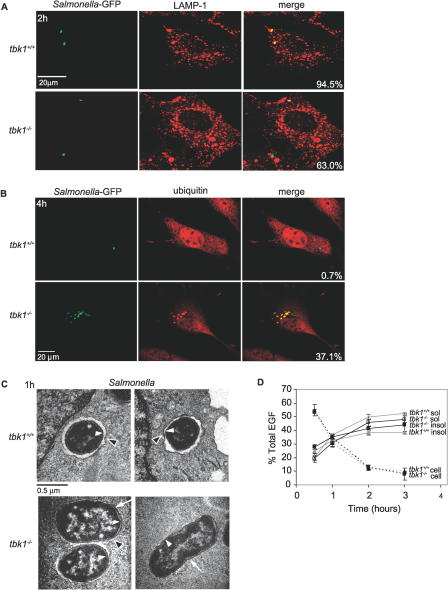
In the Absence of TBK1, Bacteria Enter the Host Cytosol (A) *Tbk1^+/+^* and *tbk1^−/−^* MEFs were infected with *Salmonella*-GFP (green). Infected cells were fixed at 2 h p.i., stained with anti-LAMP-1 antibody followed by a TRITC-labeled secondary antibody (red), and analyzed by confocal immunofluorescence microscopy. Percent colocalization was determined by counting the number of bacteria out of 150 bacteria per experiment that colocalized with LAMP-1. The experiment shown is representative of three independent experiments. (B) MEFs were infected for 1 h with the *Salmonella*-GFP (green), fixed at 4 h p.i., and stained with an anti-ubiquitin monoclonal antibody followed by a TRITC-labeled secondary antibody (red). The percent of ubiquitin-associated bacteria was determined by counting the number of bacteria out of 150 bacteria per experiment that colocalized with ubiquitin as observed by confocal microscopy. The experiment shown is representative of three independent experiments. (C) Transmission electron microscopy was performed on MEFs infected for 1 h with *Salmonella,* and images acquired at 64,000× magnification*.* White arrowheads point to bacterial membranes, black arrowheads point to host vacuolar membranes, and arrows point to bacteria no longer completely surrounded by vacuolar membranes. (D) MEFs were incubated with ^125^I-EGF and the rate of endocytic uptake (cell associated), degradation (TCA soluble), and accumulation of undegraded product (TCA insoluble) was determined at 0.5, 1, 2, and 3 h after endocytosis. The data represent the mean of four independent experiments.

Previous studies have shown that shortly after invasion, SCV colocalize with the late endosomal marker, LAMP-1 [24]. To determine the nature of the *Salmonella*-containing compartment in TBK1-deficient cells, we infected MEFs with *Salmonella-*GFP and analyzed the samples by confocal immunofluorescence microscopy using an anti-LAMP1 antibody (Figures 2A and S3). SCV in *tbk1^+/+^* cells were colocalized with LAMP-1 throughout the entire course of infection, with 94.5% colocalization at 2 h p.i. In contrast, as early as 90 min p.i. in *tbk1^−/−^* cells, individual *Salmonella* lost association with LAMP-1, and at 2 h p.i., only 63.0% of bacteria exhibited colocalization. Loss of LAMP-1 colocalization by individual bacteria was commonly observed early in infection, suggesting that replication per se was not required for this abnormal phenotype. Thus, in the absence of TBK1, many SCV lose the late endosomal marker, LAMP-1, and deviate from the characterized *Salmonella* endocytic trafficking pathway.

Because *Salmonella* in TBK1-deficient cells were found in a LAMP-1 negative compartment dispersed throughout infected cells, we reasoned that the bacteria might be in the host cytosol. It was recently reported that under circumstances when *Salmonella* was found in the cytosol, the bacteria associated with host ubiquitin [25]. Therefore, we infected MEFs with *Salmonella*-GFP and analyzed the cells by confocal immunofluorescence microscopy to determine whether bacteria were colocalized with ubiquitin (Figure 2B). In *tbk1^+/+^* cells, 0.7% of *Salmonella* colocalized with ubiquitin at 4 h p.i.; almost all of the bacteria remained in LAMP1^+^ SCV during the course of infection. In contrast, substantial numbers of *Salmonella* in *tbk1^−/−^* MEFs associated with ubiquitin (37.1% by 4 h p.i.), suggesting that *Salmonella* was released from the SCV into the cytosol. However, the possibility remained that the SCV was perforated; allowing access to cytosolic ubiquitin, but the vacuolar membrane remained around each bacterium. We directly visualized *Salmonella* in infected MEFs by transmission electron microscopy and found that by 1 h p.i. in *tbk1^−/−^* cells, 66.7% of bacteria (*n* = 30) were surrounded by vacuolar space, compared to 87.5% of bacteria (*n* = 24) in *tbk1^+/+^* cells (Figure 2C). From these data, it was not clear whether TBK1 regulated general integrity of the endocytic pathway, or whether infection specifically triggered a TBK1-dependent process. To test the general function of the endocytic compartment, we measured internalization and degradation of I^125^-labeled epidermal growth factor (EGF) in the absence of infection (Figure 2D). We would predict (based on earlier studies) that general loss of integrity of the endocytic pathway would affect lumenal pH and therefore the ability to degrade proteins taken up by endocytosis, such as EGF [30]. Endocytic uptake and processing of the radiolabeled EGF appeared similar in both *tbk1^+/+^* and *tbk1^−/−^* MEFs, demonstrating that escape of *Salmonella* into the cytosol of TBK1-deficient cells was not the result of general destabilization of the endocytic compartment. Therefore, TBK1 controls an early response to *Salmonella* infection that maintains integrity of the pathogen-containing vacuole.

### TBK1 Modulates Vacuolar Integrity in Response to Gram-Negative and Gram-Positive Bacterial Invasion

**Figure 3:**
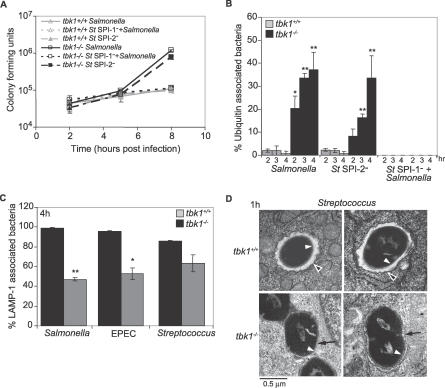
TBK1 Protects Vacuolar Integrity in Response to Bacterial Infection (A) Intracellular growth of wild-type or mutant *Salmonella* strains in *tbk1^+/+^* (triangles) and *tbk1^−/−^* (squares) MEFs was measured over time. An antibiotic resistant SPI-1-deficient strain (short dashed lines) was coinfected with an antibiotic-sensitive wild-type *Salmonella* strain to facilitate invasion by the mutant. Only antibiotic resistant colonies are shown for the SPI-1^−^ growth curve*.* (B) MEFs were infected with *Salmonella*-GFP and fixed at 2, 3, and 4 h p.i. In the case of coinfection *(St* SPI-1^−^ + *Salmonella),* the SPI-1-deficient strain was marked with GFP. The samples were stained as described in Figure 2B. Percent ubiquitin-associated bacteria in *tbk1^+/+^* MEFs (gray) or *tbk1^−/−^* MEFs (black) was calculated based on 150 bacteria counted that colocalized with ubiquitin from two to three independent experiments; * and ** denote *p* ≤ 0.05 and 0.001, respectively, according to a two-tailed Student *t*-test. (C) MEFs were infected for 1 h with the indicated bacteria and then fixed at 4 h p.i. Samples were subjected to immunofluorescence staining as described in Figure 2A. Percent LAMP-1-associated represents the number of bacteria out of 150 counted per experiment that colocalized with LAMP-1 from three independent experiments; * and ** denote *p* ≤ 0.05 and 0.001, respectively, according to a two-tailed Student *t*-test. (D) Transmission electron microscopy was performed on MEFs infected for 1 h with *Streptococcus,* and images were acquired at 64,000× magnification*.* White arrowheads point to bacterial membranes, black arrowheads point to host vacuolar membranes, and black arrows point to bacteria that are no longer completely surrounded by vacuolar membranes.

Since known mediators of TBK1-dependent signaling were not required to suppress intracellular bacterial growth, and endocytic function was not generally compromised, we hypothesized that *Salmonella* might be triggering a cellular process that requires TBK1. *Salmonella* contains two Type III secretion systems encoded on SPI-1 (termed SPI-1 T3SS) and SPI-2 (SPI-2 T3SS) that enable the bacterium to secrete proteins directly into the host cell cytosol [22,31]. The SPI-1 T3SS is required for entry into non-phagocytic cells and modulation of endosomal trafficking; later in infection, SPI-2 T3SS-dependent effectors act to regulate membrane dynamics. We tested the possibility that Type III secretion might contribute to triggering the phenotype observed in TBK1-deficient MEFs. *Salmonella* strains deficient in either the SPI-1 (*St* SPI-1^−^) or SPI-2 (*St* SPI-2^−^) encoded T3SS were assessed for their ability to replicate within MEFs and access the host cytosol (Figure 3A and 3B). The SPI-2 deficient bacteria proliferated similarly to wild-type *Salmonella* in both *tbk1^+/+^* and *tbk1^−/−^* MEFs. We also analyzed a *Salmonella* mutant lacking the SPI-2-dependent effector SifA, which exhibits defects in SCV integrity [32–34]. If SifA and TBK1 were acting in concert, we would expect *StΔsifA* to proliferate equally in *tbk1^+/+^* and *tbk1^−/−^*cells; however, we still observed increased cytosolic localization and replication by the mutant bacteria in *tbk1^−/−^* MEFs (Figure S4A and S4B). In contrast, the SPI-1-deficient bacteria (tet^R^), induced to enter independent vacuoles through bystander infection with wild-type *Salmonella* (tet^S^), as measured by tet^R^ colony-forming units or immunofluorescence, were unable to replicate in host cells of either genotype and were never released in the cytosol (Figures 3A, 3B, and S5) [35]. A double SPI-1^−^ SPI-2^−^ mutant behaved similarly to the SPI-1^−^ single mutant (unpublished data). These data suggest that a function associated with the *Salmonella* SPI-1 T3SS stimulates TBK1-dependent modulation of the pathogen-containing vacuole.

To determine whether vacuolar escape in *tbk1^−/−^*cells required a *Salmonella*-specific process, we used an invasive strain of EPEC, another Gram-negative bacterium, to infect MEFs and determined subcellular localization by quantitating colocalization with LAMP-1 (Figure 3C) [36]. LAMP-1 colocalization of EPEC was substantially decreased in *tbk1^−/−^* cells, similar to the phenotype we previously observed in *Salmonella* infection, and increased bacterial replication was observed compared to *tbk1^+/+^* MEFs (Figures 3C and S4C). We also investigated subcellular localization of *S. pyogenes,* an invasive Gram-positive bacterium, and found reproducibly that the streptococci were found less often in a LAMP-1^+^ compartment in TBK1-deficient cells, although the difference between *tbk1^−/−^* and *tbk1^+/+^* MEFs was not as striking as that observed for Gram-negative bacteria (Figure 3C). In addition, transmission electron microscopy revealed that at 1 h p.i., fewer S. pyogenes were contained in vacuoles in *tbk1^−/−^* MEFs (50%; *n* = 30) than in *tbk1^+/+^* MEFs (91.5%; *n* = 47) (Figure 3D). These results demonstrate that TBK1 is necessary for restricting Gram-negative bacteria to the endocytic compartment during infection and may also play a similar role during cellular invasion by Gram-positive bacteria.

### TBK1 Prevents Bacterial Entry into the Cytosol of Epithelial Cells and Macrophages

**Figure 4:**
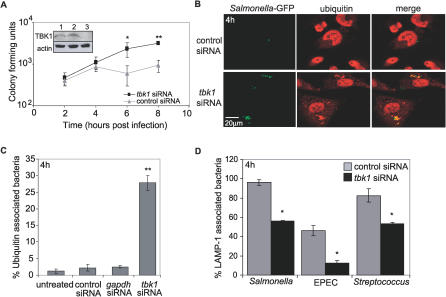
TKB1 Knockdown in Epithelial Cells Phenocopies TBK1 Deficiency in MEFs (A) HeLa cells were transfected with the indicated siRNA, and knockdown at 72 h p.i. was determined by Western blot analysis using an anti-TBK1 antibody: lane (1) no siRNA, lane (2) scrambled siRNA, lane (3) *tbk1* siRNA (inset). Blots were re-probed with anti-actin antibody as a loading control. HeLa cells were transfected with *tbk1* or control siRNA and then infected by *Salmonella* to measure intracellular growth over time; * and ** denote *p* ≤ 0.05 and 0.001, respectively, according to a one-tailed Student *t*-test comparing *tbk1* siRNA with control siRNA-treated cells at each time point. (B) HeLa cells transfected with *tbk1-*specific or control siRNA were infected with *Salmonella-*GFP. Samples were fixed at 4 h p.i., stained with anti-ubiquitin antibody followed by a TRITC-labeled secondary antibody (red), and analyzed by confocal microscopy. The experiment shown is representative of four independent experiments. (C) Quantitative analysis of cytosolic GFP-*Salmonella* was performed in infected HeLa cells left untreated or transfected with *tbk1, gapdh,* or control siRNA. Percent ubiquitin-associated represents the number of bacteria out of 150 counted per experiment that colocalized with ubiquitin at 4 h p.i. from three independent experiments; * and ** denote *p* ≤ 0.05 and 0.001, respectively, according to a two-tailed Student *t*-test comparing all samples to untreated cells. (D) HeLa cells transfected with *tbk1-*specific or control siRNA were infected for 1 h with the indicated bacteria*,* fixed at 4 h p.i., and stained with anti-LAMP1 antibody. Percent of LAMP-1-associated bacteria was determined by counting the number of bacteria out of 150 per experiment that colocalized with LAMP-1 as observed by confocal microscopy. The experiment shown is representative of at least two independent experiments; * and ** denote *p* ≤ 0.05 and 0.001, respectively, according to a two-tailed Student *t*-test comparing *tbk1* siRNA-transfected cells to control-transfected cells for each bacterial strain.

MEFs represent an amenable genetic system with which to test the role of TBK1 during bacterial infection in the absence of a gene-deficient live animal model; however, they are not a cell type that would be present during a physiological infection. To establish that loss of TBK1 caused a specific defect in response to bacterial infection, we used an RNAi approach to knock down TBK1 expression in HeLa cells, an epithelial cell line commonly used to study *Salmonella* pathogenesis, and used the treated cells to examine bacterial growth and compartmentalization. HeLa cells were transfected with *tbk1* siRNA, *gapdh* siRNA, or a nonspecific siRNA control for 72 h prior to infection; knockdown of TBK1 and GAPDH was confirmed by immunoblot analysis (Figure 4A; unpublished data). HeLa cells treated with *tbk1* siRNA supported increased replication of *Salmonella* compared to control treated cells, as observed in *tbk1^−/−^* MEFs (Figure 4A). Consistent with our previous observations in MEFs, 27.8% of bacteria in *tbk1* siRNA-treated HeLa cells were associated with ubiquitin compared to 2.2% of bacteria in cells transfected with control siRNA (Figure 4B and 4C). No significant increase in ubiquitin-associated bacteria was observed with knockdown of GAPDH. IRF3 was also knocked down by siRNA in HeLa cells; the validated knockdown had no effect on *Salmonella* growth or ubiquitin association (unpublished data). At 4 h p.i., HeLa cells transfected with *tbk1* siRNA had significantly lower numbers of *Salmonella,* EPEC, or S. pyogenes associated with LAMP-1 compared to the control transfection (Figure 4D). These data demonstrate that TBK1-dependent maintenance of vacuolar integrity is not a MEF-specific phenomenon, but also protects epithelial cells during bacterial infection.

**Figure 5:**
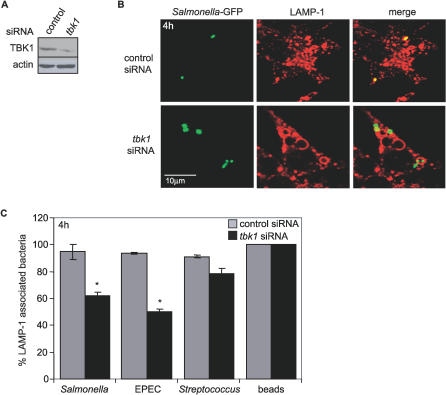
Macrophages Require TBK1 to Efficiently Restrict Bacteria in Phagosomes (A) RAW264.7 macrophages were transfected with the indicated siRNA, and knockdown at 72 h p.i. was determined by Western blot analysis using an anti-TBK1 antibody. Blots were re-probed with anti-actin antibody as a loading control. (B) RAW264.7 macrophages transfected with *tbk-*specific or control siRNA were infected with *Salmonella*-GFP, fixed at 4 h p.i., and stained with anti-LAMP1 antibody. (C) RAW264.7 macrophages transfected with *tbk-*specific or control siRNA were infected with the indicated bacterial species. Percent LAMP-1-associated bacteria or beads were determined by counting the number of bacteria or beads out of 150 per experiment that colocalized with LAMP-1 as observed by confocal microscopy. The experiment shown is representative of at least two independent experiments; * denotes *p* ≤ 0.05, using a two-tailed Student *t*-test comparing *tbk1* siRNA-transfected cells to control-transfected cells.

We additionally sought to determine if there was a requirement for TBK1 in immune effector cells such as macrophages. We used RNAi to knock down TBK1 expression in the RAW264.7 macrophage cell line (Figure 5A). It was previously reported that *Salmonella* grow poorly in macrophage cytosol, so LAMP-1 colocalization was assessed to reflect escape of bacteria from the SCV [32]. At 4 h p.i., in macrophages treated with *tbk1* siRNA, only 62.0% of *Salmonella* colocalized with LAMP-1 compared to 94.7% colocalization in control siRNA-treated cells (Figure 5B and 5C). *Tbk1* siRNA treatment of RAW264.7 cells also resulted in decreased LAMP-1 colocalization with bacteria during infection by EPEC or *S. pyogenes.* In contrast, 2-μm beads taken up by phagocytosis remained completely associated with LAMP-1 in both *tbk1* and control siRNA-treated cells. From these observations, we conclude that TBK1 regulates the integrity of pathogen-containing vacuoles in multiple cell types.

## Discussion

We have shown here that the IKK-like kinase, TBK1, mediates an early cellular response to bacterial infection. In the absence of TBK1, *Salmonella* replicated rapidly and to high levels in MEFs. The IRF3-Type I inteferon axis which contributes to antiviral immunity was not required for the growth restrictive function of TBK1, nor was de novo transcription or translation. After entry into *tbk1^−/−^*cells, *Salmonella* escaped into the cytosol where proliferation occurred. Loss of vacuolar integrity in TBK1-deficient cells was not specific to *Salmonella* infection, but occurred during infection by Gram-negative and Gram-positive pathogenic bacteria. Thus, TBK1 protects cells during bacterial infection by confining invading pathogens to a membrane-bound compartment.

The best studied TBK-dependent signaling pathway triggered by bacterial infection is LPS-mediated induction of Type I interferons through TLR4, TBK1, and IRF3 [9,11,37,38]. However, our data showed that TBK1 did not require IRF3 or Type I interferon to exert a protective effect on host cells during bacterial infection. It is yet unclear whether TLR4 activation contributes to TBK1-dependent maintenance of vacuolar integrity. We still observed a protective effect by TBK1 on pathogen-containing vacuoles in HeLa cells, which do not express surface TLR4 due to lack of the accessory protein, MD2 [39]. This observation suggests that TLR4 signaling is not absolutely required for the restrictive function of TBK1 in bacterial infection, but further studies will be necessary to definitively determine the role of TLR4 or other TLRs in modulation of vacuolar integrity by TBK1.

The vacuolar compartment is a restrictive antimicrobial environment because of its ability to decrease pH, produce degradative enzymes, and in some cell types, to generate harmful reactive oxygen species in a confined environment [40–42]. In contrast, there are few cytosolic antimicrobial mechanisms, possibly to minimize damage to cytosolic host machinery. Given the more permissive nature of the host cytosol, it is surprising that relatively few bacterial pathogens exploit this intracellular niche [43]. TBK1-dependent mechanisms may prevent many bacterial pathogens from accessing the cytosol by modulating integrity or function of the endocytic compartment during infection. Our data suggest two nonexclusive models by which TBK1 may contribute to the cellular response to bacterial invasion. First, TBK1 could function at the post-transcriptional level in response to an infection by phosphorylating target proteins. In the case of bacterial infection, there may be TBK1 kinase substrates whose function directly or indirectly modifies the pathogen-containing vacuole. Secondly, TBK1 may act prior to infection to establish a state of immune competence, perhaps by regulating expression of gene products that are important for the immediate response to bacterial invasion. This model requires that TBK1 have some constitutive activity prior to infection. Since there is a known requirement for TBK1 in embryonic development in the absence of infection, it is likely that TBK1 may function in normal adult animals in the absence of infection as well [5–7]. Indeed, two recent studies have identified TBK1 as a regulator of angiogenesis and oncogenesis [44,45]. Furthermore, microarray analysis of uninfected *tbk1^+/+^* and *tbk1^−/−^* MEFs identified over 400 genes that were differentially expressed, some of which have known associations with innate immune function (ALR and MXDO, unpublished data).

At the molecular level, there are at least two mechanisms by which TBK1 may ultimately regulate vacuolar integrity. In response to bacterial invasion, TBK1 could control initiation of autophagy, which can capture bacteria in damaged vacuoles [46,47]. This possibility is less likely, as colocalization with LC3, a marker of autophagosomes, was slightly higher in infected *tbk* ^−/−^ cells than wild-type cells. However, it is notable that all of the bacterial species, for which we showed cytosolic localization in *tbk1^−/−^* cells, have mechanisms by which vacuolar membranes are damaged during infection, i.e., *Salmonella* and EPEC contain Type III secretion machinery, and S. pyogenes encodes the pore-forming toxin Streptolysin O [48–50]. Alternatively, a TBK1-dependent target could modulate influx/efflux of ions or water into the pathogen-containing vacuole to maintain its physical continuity or otherwise alter membrane dynamics in response to infection. Previous studies have demonstrated that host cells activate repair mechanisms in response to membrane damage [51,52]. Our findings are consistent with damage to the vacuolar membrane as a possible trigger for TBK1-dependent function during bacterial infection.

The innate immune system is required both for controlling pathogen replication and for communicating with other cell types. In viral infections, TBK1 clearly acts as a regulator of innate immunity by communicating with other cells through Type I interferon, which contributes to control of viral replication. However, we have demonstrated that in bacterial infections, TBK1 plays an important role in limiting pathogen replication by protecting the integrity of the pathogen-containing vacuole, independently of Type I interferon. Our findings do not preclude an additional requirement for TBK1 in antibacterial immunity through stimulation of cytokine or chemokine expression, but suggest an early TBK1-dependent mechanism by which host cells can achieve innate control of bacterial infection.

## Supporting Information

Figure S1 Trafficking of *Salmonella* Populations in *tbk1^−/−^* MEFs (A) *Tbk1^+/+^* and *tbk1^−/−^* MEFs were transfected with a LC3-GFP-expressing plasmid (green) to identify autophagosomes and infected with *Salmonella*-RFP (red). Infected cells were fixed at 1 h p.i. and analyzed by confocal microscopy. Percent colocalization was determined by counting how many bacteria out of 100 per experiment that colocalized with LC3-GFP (*n* = 3). White arrowheads point to *Salmonella* colocalized with LC3-GFP, white outlined arrowheads point to bacteria not colocalized with LC3-GFP.
(B) *Tbk1^+/+^* and *tbk1^−/−^* MEFs were treated with Lysotracker (a dye that fluoresces in acidic compartments) and infected with *Salmonella*-GFP (green). Infected cells were fixed 1 h p.i. and analyzed by confocal microscopy. Percent colocalization was determined by counting the number of bacteria out of 150 bacteria that colocalized with bright red acidic compartments.
(10 MB PDF)

Figure S2 Validation of rIFN-β Stimulation and α-Amanitin Inhibition in MEFs Infected with *Salmonella*
(A) Quantitative RT-PCR was performed to determine *mx1* induction in uninfected, 1 h, and 8 h *Salmonella*-infected MEFs in the presence of 100 U/ml rIFN-β normalized to uninfected untreated cells. ** denotes *p* ≤ 0.001, according to a two-tailed Student *t*-test comparing rIFN-β-treated samples to an untreated sample of the same genotype.
(B) *Salmonella* intracellular growth curve in *tbk1^+/+^* (gray triangles) and *tbk1^−/−^* (black squares) MEFs was measured over time in the absence (solid lines) or presence (dashed lines) of the RNA polymerase II inhibitor, α-amanitin. Where indicated, MEFs were treated with 50 μg/ml α-amanitin 1 h prior to and during infection. The data shown are representative of three independent experiments.
(C) Quantitative RT-PCR was performed to assess *ip10* induction in uninfected and 8 h *Salmonella-*infected *tbk1^+/+^* MEFs in the presence or absence of α-amanitin (50 μg/ml). ** denotes *p* ≤ 0.001, according to a two-tailed Student *t*-test comparing infected untreated and α-amanitin-treated cells to uninfected cells.
(271 KB PDF)

Figure S3 Kinetic Analysis of LAMP-1 Colocalization with *Salmonella* in *tbk1^+/+^* and *tbk1^−/−^* MEFs *Tbk1^+/+^* and *tbk1^−/−^* MEFs were infected with *Salmonella*-GFP (green). Infected cells were fixed at 2, 4, and 6 h p.i., stained with anti-LAMP-1 antibody followed by a TRITC-labeled secondary antibody (red), and analyzed by confocal immunofluorescence microscopy. Percent colocalization was determined by counting the number of bacteria out of 150 bacteria per experiment that colocalized with LAMP-1. The experiment shown is representative of three independent experiments.
(7.2 MB PDF)

Figure S4 A *Salmonella sifA* Mutant Strain and EPEC Display Similar Growth and Vacuolar Escape as Wild-Type *Salmonella* in *tbk1^−/−^* MEFs (A) MEFs were infected for 1 h with the indicated bacterial strains and then fixed at 2, 3, or 4 h p.i. Results from *tbk1^+/+^* MEFs are shown in gray bars and *tbk1^−/−^* MEFs are shown in black bars. Samples were stained as in Figure 4B. Percent ubiquitin-associated represents the number of bacteria out of 150 counted per experiment that colocalized with ubiquitin from two to three independent experiments. * and ** denote *p* ≤ 0.05 and 0.001, respectively, according to a two-tailed Student *t*-test comparing each time point of *tbk1^−/−^* to *tbk1^+/+^* for each bacterial strain.
(B) Intracellular growth of wild-type *Salmonella* (*St;* solid lines) or a *ΔsifA* mutant strain (*StΔsifA;* dashed lines) was measured over time in *tbk1^+/+^* (triangles) and *tbk1^−/−^* (squares) MEFs. The data are representative of four independent experiments.
(C) Intracellular growth of EPEC was measured over time in *tbk1^+/+^* (triangles) and *tbk1^−/−^* (squares) MEFs. * denotes a *p-*value of ≤ 0.05, according to a one-tailed Student *t*-test comparing *tbk1^−/−^* to *tbk1^+/+^* at each time point. The data are representative of three independent experiments.
(273 KB PDF)

Figure S5 A *Salmonella* SPI-1 Mutant Remains in LAMP-1^+^ Vacuoles in *tbk1^+/+^* and *tbk1^−/−^* MEFs throughout Infection (A) *Tbk1^+/+^* and *tbk1^−/−^* MEFs were infected with *St* SPI-1^−^-GFP (green) in the presence of wild-type *Salmonella* to induce bystander uptake. Infected cells were fixed at 4 h p.i., stained with DAPI (blue) and an anti-LAMP-1 antibody, followed by a TRITC-labeled secondary antibody (red) prior to analysis by confocal microscopy. Percent colocalization was determined by counting the number of GFP^+^ bacteria (SPI-1^−^) out of 150 GFP^+^ bacteria colocalized with LAMP-1. The data are representative of three independent experiments.
(B) *Tbk1^+/+^* and *tbk1^−/−^* MEFs were treated with Lysotracker, a dye that fluoresces brightly in acidic compartments, and subsequently infected with *St* SPI-1^−^-GFP (green) in the presence of wild-type *Salmonella* to induce bystander uptake. Infected cells were fixed 1 h p.i. and analyzed by confocal microscopy.
(C) MEFs were treated with Lysotracker, infected with either wild-type *Salmonella-*GFP or *St* SPI-1^−^-GFP in the presence of wild-type unmarked *Salmonella (Salmonella)* to induce bystander uptake for 1 h and fixed at 1 h and 4 h p.i. Percent Lysotracker-associated bacteria was determined by counting the number of GFP^+^ bacteria out of 150 GFP^+^ bacteria per experiment that colocalized with bright red acidic compartments as observed via confocal microscopy (*n* = 3).
(9.4 MB PDF)

Table S1 Bacterial Strains Used in This Study (17 KB PDF)

### Accession Numbers

The DNA sequences used for primer design in this study from the NCBI Entrez Nucleotide sequence database (http://www.ncbi.nlm.nih.gov/entrez/query.fcgi?db=Nucleotide) are *actin* (NM_007393), *ip10* (NC_00071), *mx1* (NM_010846), and *tbk1* (NC_00076.4).

## Abbreviations

EGF: epidermal growth factor
EPEC: enteropathogenic *Escherichia coli;*
IRF3: interferon regulatory factor-3
LAMP-1: lysosomal-associated membrane protein 1
LPS: lipopolysaccharide
MEF: murine embryonic fibroblast
m.o.i.: multiplicity of infection
p.i.: post-infection
TBK1: TANK-binding-kinase-1
TLR: toll-like receptor
T3SS: Type III secretion system
SCV: *Salmonella*-containing vacuole
SPI-1: *Salmonella* pathogenicity island-1
