# Supplementary material for: TBK1 Protects Vacuolar Integrity during Intracellular Bacterial Infection
Source: PLoS Pathog. 2007 Mar 2;3(3):e29. doi: 10.1371/journal.ppat.0030029 (PMC1808071; doi:10.1371/journal.ppat.0030029)

Supplemental Figure 1

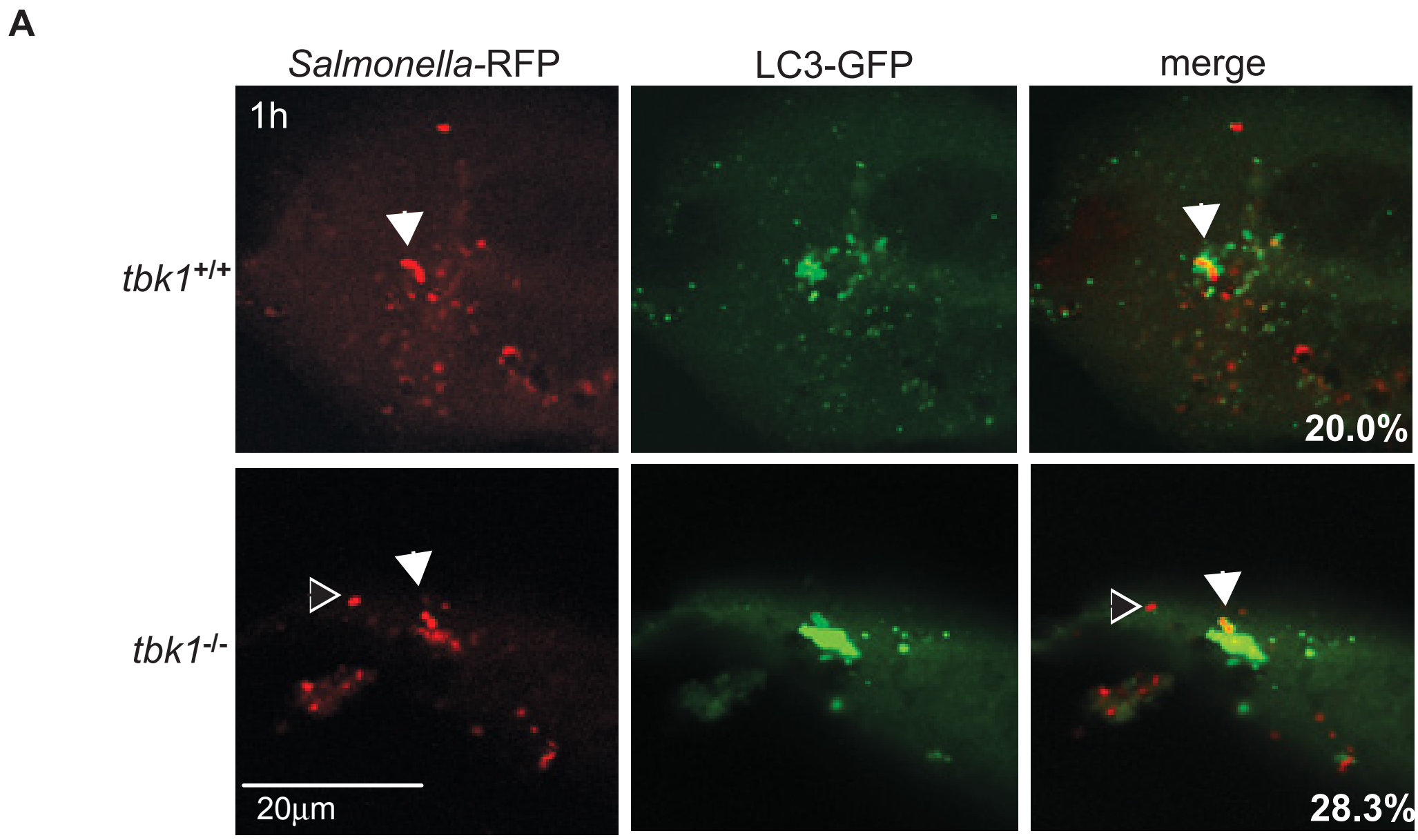

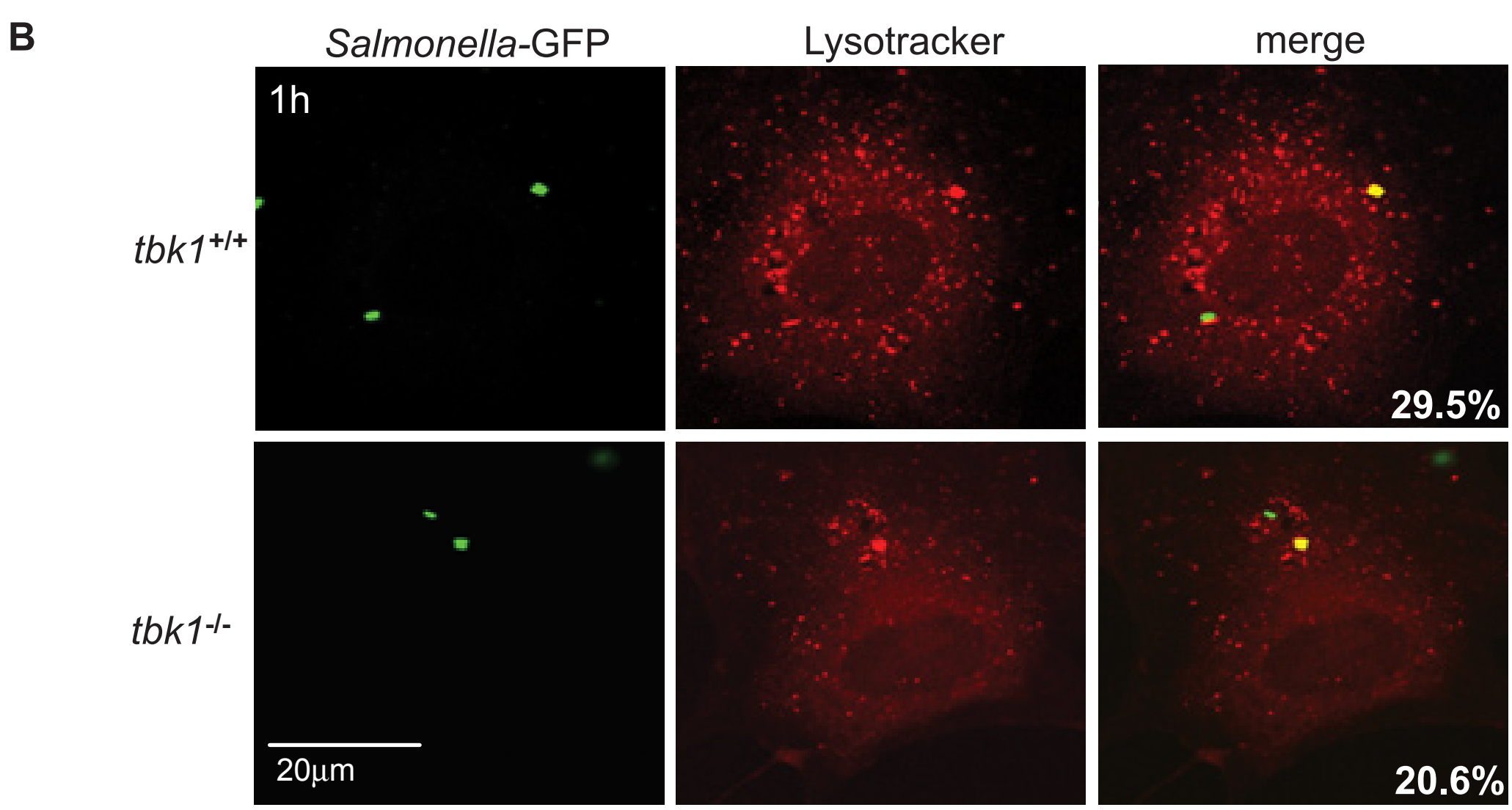

Supplement: Supplementary file 1 [file ppat.0030029.sg001.pdf]
