# Supplementary material for: TBK1 Protects Vacuolar Integrity during Intracellular Bacterial Infection
Source: PLoS Pathog. 2007 Mar 2;3(3):e29. doi: 10.1371/journal.ppat.0030029 (PMC1808071; doi:10.1371/journal.ppat.0030029)
Supplement: Supplementary file 6 [file ppat.0030029.st001.pdf]

Table S1. Bacterial strains used in this study.

Name	Genotype or Phenotype	Strain	Reference	Stock #
Salmonella enterica serovar Typhimurium	wildtype – virulent in mice	SL1344	[1]	MOR-23
Salmonella-GFP	wildtype + pFPV25.1	SL1344	[2]	MOR-36
Salmonella-RFP	wildtype + pCR-BluntII-Topo-RFP	SL1344	This study	MOR-149
St SPI-1^{-}-GFP	*orgA::Tn5lacZY* + pFPV25.1	SL1344	[3];this study	MOR-37
St SPI-2^{-}	*ssaV::MudJ*	SL1344	[4]	MOR-25
St SPI-2^{-}-GFP	*ssaV::MudJ* + pFPV25.1	SL1344	[4];this study	MOR-38
St ΔsifA	*ΔsifA*	SL1344	[5]	MOR-26
St ΔsifA-GFP	*ΔsifA* + pFPV25.1	SL1344	[5];this study	MOR-38
EPEC	invasive	E2348/69	[6]	MOR-180
Streptococcus pyogenes	invasive	MGAS 166	[7]	MOR-113

References

1. Wray C, Sojka WJ (1978) Experimental Salmonella typhimurium infection in calves. Res Vet Sci 25: 139-143.
2. Valdivia RH, Falkow S (1996) Bacterial genetics by flow cytometry: rapid isolation of Salmonella typhimurium acid-inducible promoters by differential fluorescence induction. Mol Microbiol 22: 367-378.
3. Jones BD, Falkow S (1994) Identification and characterization of a Salmonella typhimurium oxygen-regulated gene required for bacterial internalization. Infect Immun 62: 3745-3752.
4. Suvarnapunya AE, Stein MA (2005) DNA base excision repair potentiates the protective effect of Salmonella Pathogenicity Island 2 within macrophages. Microbiology 151: 557-567.
5. Stein MA, Leung KY, Zwick M, Garcia-del Portillo F, Finlay BB (1996) Identification of a Salmonella virulence gene required for formation of filamentous structures containing lysosomal membrane glycoproteins within epithelial cells. Mol Microbiol 20: 151-164.
6. Donnenberg MS, Calderwood SB, Donohue-Rolfe A, Keusch GT, Kaper JB (1990) Construction and analysis of TnphoA mutants of enteropathogenic Escherichia coli unable to invade HEp-2 cells. Infect Immun 58: 1565-1571.
7. Musser JM, Kapur V, Szeto J, Pan X, Swanson DS, et al. (1995) Genetic diversity and relationships among Streptococcus pyogenes strains expressing serotype M1 protein: recent intercontinental spread of a subclone causing episodes of invasive disease. Infect Immun 63: 994-1003.
